# Public health interventions for the prevention and management of overweight and obesity in children and adolescents: a rapid review

**DOI:** 10.3389/fpubh.2026.1893709

**Published:** 2026-09-08

**Authors:** Ketaki Chandiok, Archi Bafna Kothari, Bharati Kulkarni, Ranadip Chowdhury, Mahesh Kumar Mummadi, Samarasimha N. Reddy, SubbaRao M. Gavaravarapu, Zaozianlungliu Gonmei, Aparna Mukherjee, Priyanka Gupta Bansal

**Affiliations:** 1 Division of Reproductive, Child Health and Nutrition, Indian Council of Medical Research, New Delhi, India; 2 Indian Council of Medical Research - National Institute of Nutrition, Hyderabad, India; 3 Society for Applied Studies, New Delhi, India

**Keywords:** adolescents, children, management, obesity, overweight, prevention

## Abstract

**Background:**

Overweight and obesity among children and adolescents have become major global public health concerns. Given the critical role of childhood and adolescence in the development of lifelong health behaviors, identifying effective intervention strategies for obesity prevention and management is essential. This rapid review aimed to synthesize evidence from systematic reviews evaluating interventions for the prevention and management of overweight and obesity among children and adolescents.

**Methods:**

The review protocol was registered with PROSPERO (CRD420261281992) and conducted in accordance with PRISMA 2020 guidelines. A literature search of the PubMed database was performed to identify systematic reviews and meta-analyses published between January 2015 and 29 July 2026. Eligible reviews included interventions targeting children and adolescents aged 0–19 years. Methodological quality was assessed using AMSTAR 2. Findings were synthesized narratively according to intervention type and categorized as prevention, management, or combined approaches.

**Results:**

Of the 765 records identified, 77 systematic reviews, including 51 meta-analyses, met the inclusion criteria. Interventions included dietary, physical activity, behavioral/educational, digital health, nutraceutical, and multicomponent approaches. Multicomponent interventions integrating dietary, physical activity, and behavioral strategies demonstrated the most consistent effectiveness in improving anthropometric outcomes among children and adolescents. Family involvement, school-based delivery, and interventions initiated during early childhood appeared to enhance effectiveness. In contrast, single-component interventions showed mixed or limited effects. Digital health interventions demonstrated variable effectiveness, while evidence related to environmental and policy-level determinants remained limited. Methodological assessment indicated that most included reviews were of low or critically low quality, with only seven reviews rated as high quality.

**Conclusion:**

Multicomponent interventions combining dietary, physical activity, and behavioral strategies appear to be the most effective approaches for preventing and managing overweight and obesity among children and adolescents. However, substantial methodological limitations and heterogeneity across studies warrant cautious interpretation of findings. Future research should prioritize rigorous, high-quality multicomponent studies incorporating environmental and contextual determinants to support sustainable and scalable public health interventions.

## Introduction

Overweight and obesity have emerged as major public health concerns worldwide (1), characterized by a complex interplay of biological, behavioral, and environmental factors (2). Over the past few decades, obesity prevalence has increased substantially across the globe, with rates among children and young people (aged 5–24 years) nearly tripling between 1990 and 2021 (3). According to the World Health Organization, in 2022, more than 390 million children and adolescents aged 5–19 years in the world were living with overweight, including approximately 160 million with obesity (4). Early onset obesity is associated with an increased risk of developing cardio-metabolic conditions with adverse effects often persisting into adulthood (5).

In India, the burden has increased across age groups and geographic regions, reflecting the country’s growing dual burden of malnutrition. As per National Family Health Survey-5 (NFHS-5; 2019–21), the prevalence of overweight among children under 5 years increased from 2.1% in NFHS-4 (2015–16) to 3.4% in NFHS-5 (6, 7). Similarly, among adolescents aged 15–19 years, the prevalence of overweight increased from 4.2 to 5.4% among females and 4.8 to 6.6% among males between the two survey rounds.

Childhood and adolescence represents critical development periods for establishment of self-regulatory behaviors through continuous interaction with parents, teachers, mentors and peers. Such behaviors play a fundamental role in promoting health and well-being across the life course (8). Consequently, interventions targeting these age groups are likely to yield long-term benefits for health and well-being while also reducing the direct and indirect economic burden associated with obesity (9).

Although numerous studies and reviews have examined strategies for preventing and managing obesity among children and adolescents, the evidence remains difficult to interpret collectively because of the limited synthesis across intervention levels and settings. Interventions vary considerably in design, implementation approaches, and outcome measures, often without sufficient consideration of development stages and contextual influences. As a result, it remains unclear which interventions are most effective, for whom, and under what circumstances. This lack of clarity poses challenges for translating evidence into scalable and sustainable public health strategies. Furthermore, the rising burden of childhood obesity across diverse settings, together with lifestyle changes following the COVID-19 pandemic, underscores the need for a timely and policy-relevant synthesis of the available evidence.

In this context, the present rapid review synthesizes evidence from systematic reviews and meta-analyses to provide a comprehensive overview of interventions aimed at preventing and managing overweight and obesity among children and adolescents. The review seeks to identify effective intervention approaches and highlight key evidence gaps to inform future research, context-specific program design, school health initiatives, and public health policy planning.

## Materials and methods

The review adheres to the Preferred Reporting Items for Systematic Reviews and Meta-analyses 2020 guidelines (10). It was registered with the *International Prospective Register of Systematic Reviews* (PROSPERO; Registration Number: CRD420261281992) (11).

### Search strategy

The Pubmed database was searched to identify eligible systematic reviews (and/or meta-analyses) published between January 2015 to 29 July 2026. Studies conducted among human population, available in full text and published in English language were considered. The search strategy is provided in the Supplementary data sheet 1. Given the time-sensitive nature of the review, a streamlined search strategy was employed to identify relevant articles.

### Eligibility criteria

The eligibility requirements for the included systematic reviews were: systematic Reviews (and/or meta-analyses) of randomized controlled trials, quasi-experimental, and pre-post study designs evaluating public health interventions for the prevention and/or management of overweight and/or obesity in children and adolescents aged 0 and 19 years. Outcomes of interest included: a change in weight, waist circumference, waist-to-hip ratio, BMI, BMI percentile, BMI z-score, body fat percentage, skin-fold thickness. To ensure methodological rigor, only systematic reviews (and/or meta-analyses) that conducted a comprehensive literature search in at least three electronic databases, employed a paired independent review, and performed a risk of bias assessment were included. Reviews focusing on pharmacological (medication-based prevention/ treatment, drug trials) and/or surgical procedures or invasive medical treatment were excluded from this review.

### Screening and data extraction

The retrieved articles were managed using the Rayyan screening software.^1^ For preliminary screening, two reviewers (KC and ABK) independently performed the searches, screened the titles and abstracts to identify potentially relevant articles. Subsequently, the two reviewers assessed the full-text articles for final inclusion to identify systematic reviews and/or meta-analyses in accordance with the pre-defined eligibility criteria. Any disagreements between the reviewers were resolved through discussion, and where necessary, through consultation with a third reviewer (PGB) until consensus was achieved.

### Quality assessment

The methodological quality of the included systematic reviews was assessed using *A Measurement Tool to Assess Systematic Reviews 2* (AMSTAR 2). The tool evaluates 7 critical and 9 non-critical domains. Each domain is rated as “Yes,” “Partial Yes,” or “No” depending on the extent to which the review satisfies the assessment criteria. Based on AMSTAR 2 guidance, reviews were classified as high quality (no or one non-critical weakness), moderate quality (more than one non-critical weakness), low quality (one critical flaw with or without non-critical weaknesses), or critically low quality (more than one critical flaw with or without non-critical weaknesses) (12). Two reviewers (KC and ABK) independently assessed methodological quality using the AMSTAR 2 tool. Any discrepancies were resolved through discussion with a third reviewer (PGB) until consensus was reached.

### Data synthesis

The findings of the included reviews were synthesized narratively. Information pertaining to type of intervention, first author, year, study type, study population, intervention category (prevention, management or both), intervention details, comparator/control, outcome assessed, intervention duration, quality of the study, study years, effect size, remarks, databases searched for the review, number of studies reviewed, sample size of all the included studies, countries/region, represented, intervention setting, strengths & limitations were collated in the study characteristics table (as annexed in Supplementary Table 1). A narrative synthesis was employed owing to the heterogeneity of intervention administered, populations employed, and outcomes assessed. The key findings of the included reviews were narratively synthesized by intervention type and further categorized based on whether the intervention addressed prevention, management, or both.

## Results

**Figure 1:**
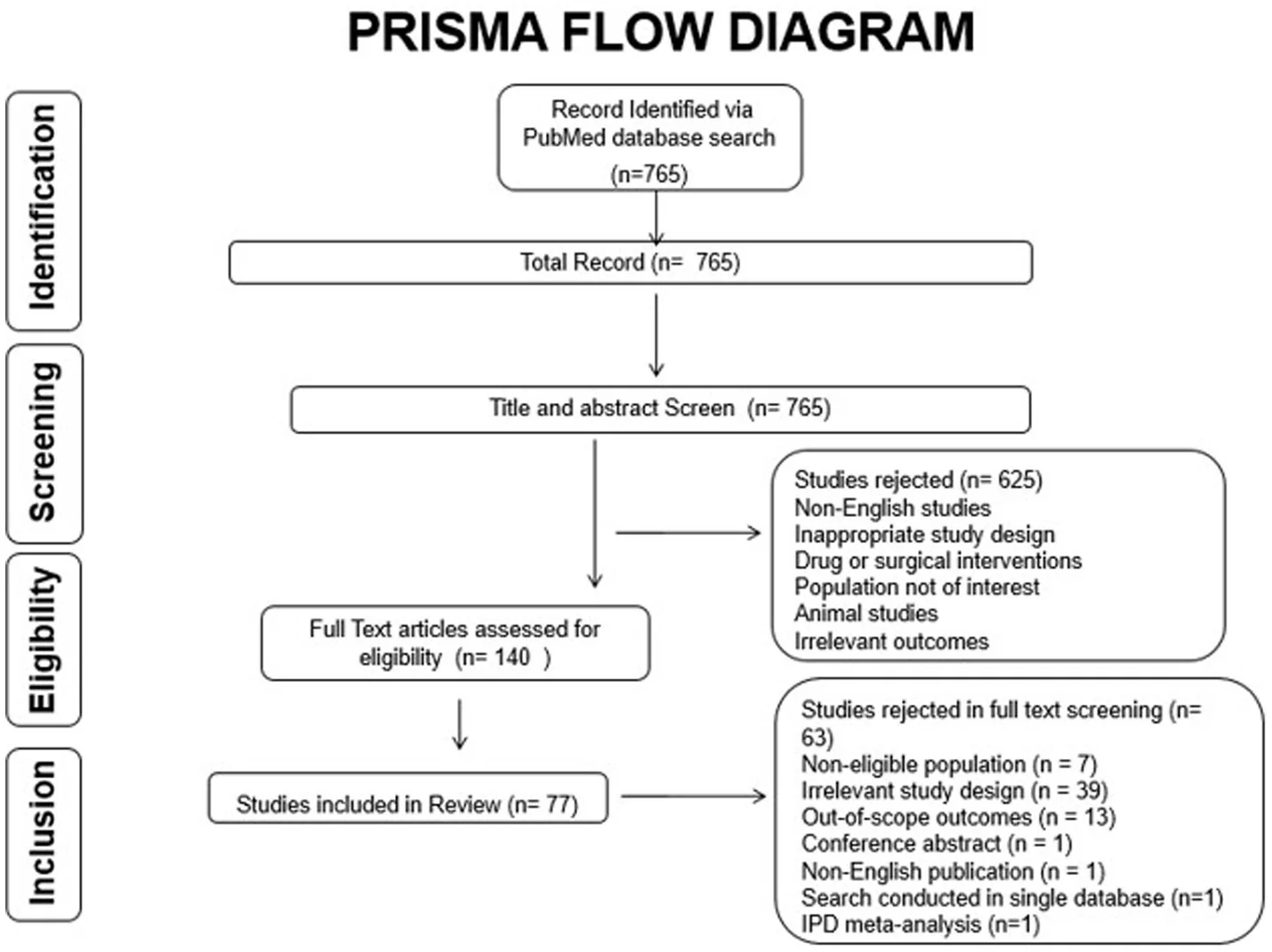
PRISMA Flow chart summarizing the identification and selection of studies for systematic review.

A total of 765 articles were retrieved from Pubmed search. After screening for titles and abstract, 140 articles underwent full-text review and were assessed for eligibility in accordance with the predefined inclusion and exclusion criteria. After the review of the full-text articles, 63 articles were excluded. The reasons for exclusion included irrelevant study design (*n* = 39), outcomes outside the scope of the review (*n* = 13), non-eligible population (*n* = 7), search conducted in a single database, individual participant data meta-analysis, conference abstract and non-English publication (*n* = 1 each). The PRISMA flow chart is provided in the Figure 1.

**Figure 2:**
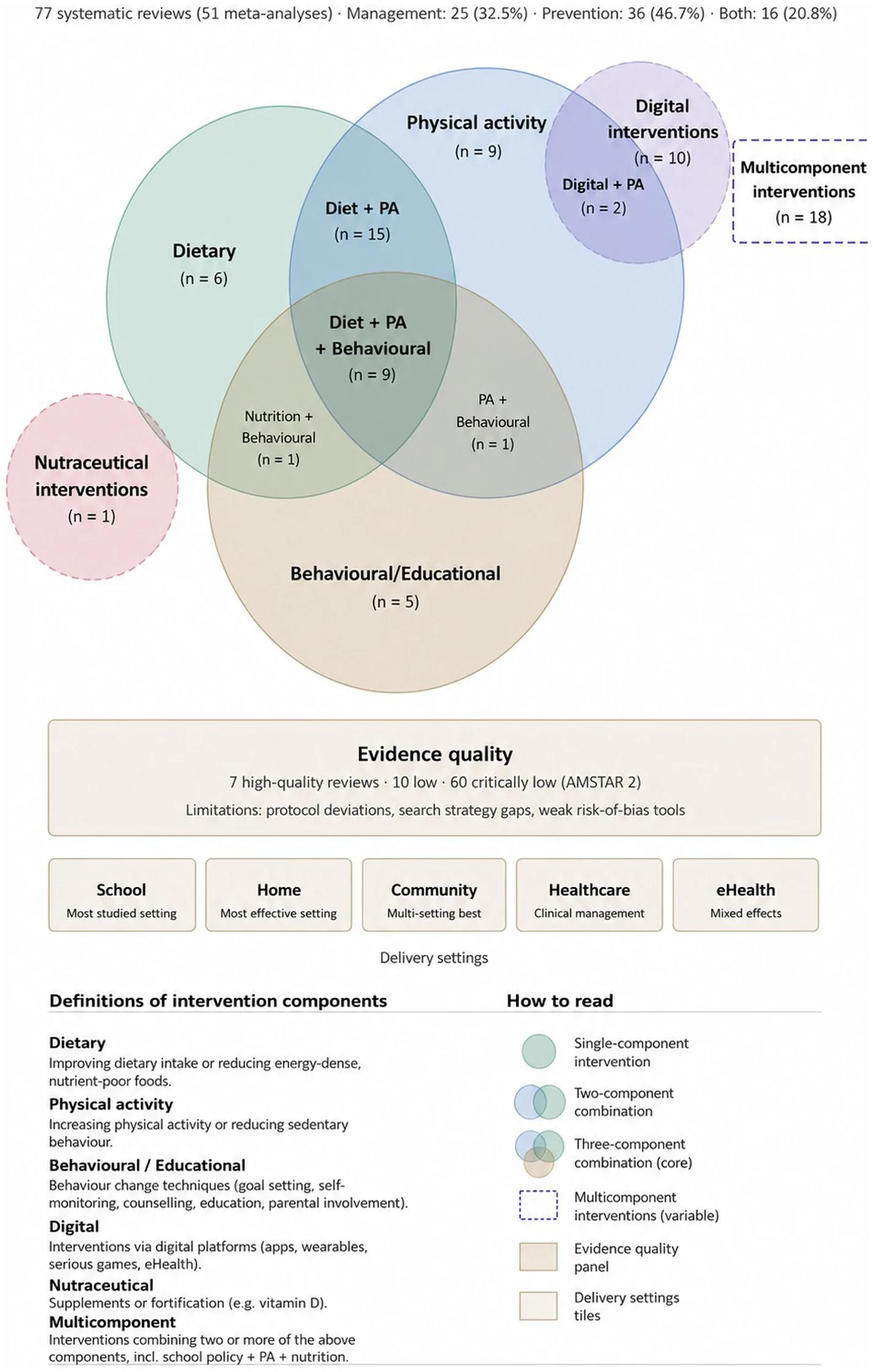
Conceptual framework of intervention components for prevention and management of overweight and obesity.

A total of 77 systematic reviews (including 51 meta-analyses) were reviewed further. Among these, 25 reviews focused on management (32.5%), 36 reviews focused on prevention (46.7%), and 16 reviews focused on both management and prevention (20.8%). The interventions evaluated pertained to dietary (*n* = 6, 7.8%), physical activity (*n* = 9, 11.7%), digital health (*n* = 10, 13%), educational/behavioral (*n* = 5, 6.5%) and nutraceutical (*n* = 1, 1.3%) interventions. In addition to it, the combination of interventions included dietary and physical activity (*n* = 15, 19.5%), digital health and physical activity (*n* = 2, 2.6%), behavioral and nutrition interventions (*n* = 1, 1.3%), physical activity and behavioral (*n* = 1, 1.3%), dietary, physical activity and behavioral (*n* = 9, 11.7%) and multicomponent interventions (*n* = 18, 23.3%). The characteristics of the interventions for all reviews are described in Supplementary Table 1. A conceptual framework of the interventions identified has been provided in Figure 2.

Multicomponent interventions consistently showed the most favorable effects. These interventions, typically combining dietary, physical activity, and/or behavioral components as demonstrated by systematic reviews (13–15) and meta-analytical findings across a range of anthropometric measures (16–21). School-based programs were particularly effective in some (16, 18, 22–25) but not all reviews reported (26). Parental or family involvement and longer intervention duration were associated with greater effectiveness (27–29). Multicomponent interventions also demonstrated consistent, though smaller, improvements across cardio-metabolic outcomes, including lipid profiles, blood pressure, and fasting insulin levels (17).

Dietary, physical activity and behavioral interventions demonstrated mixed results, with some (30–34) but not all (35, 36) studies reporting significant effects. Findings from school based interventions demonstrated inconclusive effects on BMI (37). Interestingly, Mast et al. (38) demonstrated sex based differential responses to lifestyle interventions. Whitehead et al. (39) highlighted the important role of nurses as key stakeholders for the delivery of childhood obesity interventions and recommended their greater involvement.

Further, combined dietary and physical activity interventions were more effective than either intervention alone (40–42), especially in younger children (≤5 years) (43), but not adolescents (44). Further, dietary and physical activity interventions implemented for the management of overweight or obesity were effective in improving BMI-related outcomes (45, 46). Combined diet and physical activity interventions were reported to be more effective than diet-only interventions in improving waist circumference, triglycerides, and overall body composition (45). Evidence suggested diminishing effectiveness with increasing age, with limited or no significant impact observed among adolescents (47). In contrast to this, systematic reviews by Adom et al. (48) and Hayba et al. (49) reported inconsistent findings among African and ethical and racial minorities, respectively. Likewise, Obita and Alkhatib (50) reported non-significant effects of dietary and physical activity interventions on anthropometric outcomes. School- and home-based settings were identified as suitable setting for intervention (51–53). Importantly, Frerichs et al. (54) noted the importance of involving children and youth in participatory roles.

Physical activity interventions demonstrated effectiveness in improving anthropometric outcomes. Interventions incorporating both aerobic and resistance training (55–57), as well as high-intensity or mixed exercise formats (58, 59), resulted in greater reductions in body mass, fat mass, and BMI compared to aerobic exercise alone. On the other hand, Yan et al. (60) reported benefits of adherence to aerobic exercise in improving body composition measures as compared those following usual control. Physical activity interventions (56, 57) resulted in significant improvements in lipid profiles and cardiovascular parameters, including reductions in low-density lipoprotein cholesterol, total cholesterol, triglycerides, and blood pressure, along with increases in high-density lipoprotein cholesterol. These effects were more pronounced among adolescents. However, some high-quality reviews reported minimal or no clinically meaningful changes in BMI despite improvements in physical fitness (61). Further, Yuksel et al. (62) highlighted the importance of school-based interventions in promoting physical activity and fitness. Caner and Erdoğan Yüce (63) demonstrated the effectiveness of yoga in reducing BMI and body fat percentage in children and adolescents with obesity.

Dietary interventions alone resulted in modest reductions in BMI and obesity prevalence (64, 65). Exclusive breastfeeding (66) was associated with more favorable BMI trajectories as compared to formula-fed or mixed-fed. Early-life interventions (67) among children aged 6 months to 6 years had a positive effect in child diet quality. Manapurath et al. (68) reported limited impact of protein quantity or quality during complementary feeding on growth or body composition. Nonguierma et al. (69) emphasized the importance of school-based settings in improving dietary intake, anthropometric or biometric measures, and physical activity outcomes.

Digital health interventions showed mixed effects. Interventions involving wearable devices (70), active video games (71, 72), game based (73), technology-based interventions with parental involvement (74) or mobile health applications (75) demonstrated reductions in BMI measures Parental involvement appeared to enhance effectiveness. In contrast to this, Liu et al. (76) reported the effectiveness of serious games in increasing physical activity, but not on body composition score and dietary habits improvements. Hammersley et al. (77) and Yien et al. (78) reported no significant effects of eHealth-based interventions and mobile health technology-based interventions on among children and adolescents with obesity risks. Turner et al. (79) noted limited effects of mobile and wireless technologies on outcomes such as body mass index, physical activity, breakfast and fruit/vegetable intake. Digital tools that promoted physical activity improved physical fitness and resulted in significant improvements in BMI and body fat among both children and adolescents (80, 81).

Educational and behavioral interventions (82–84) alone demonstrated limited or non-significant effects on BMI outcomes, in some but not all cases (85, 86), mainly owing to the difference in the behavioral intervention studied. Redsell et al. (87) noted that a combination of nutrition and behavioral interventions comprising of diet and responsive feeding focused interventions delivered to pregnant women and parents of children <2 years of age are promising strategies for obesity prevention and also suggested for improvement in parental feeding practices like infant diet and responsiveness to infant cues. López-Alarcón et al. (88) reported a lack of evidence on the efficacy and safety of yoga and mindfulness therapy for the management of obesity among children and adolescents in context of the combination of physical activity and behavioral interventions delivered. Nutraceutical interventions, such as vitamin D supplementation (89), demonstrated small reductions in BMI during early childhood.

Seven systematic reviews met the criteria for the high methodological quality (30, 41, 43, 47, 61, 64, 67) 10 studies were rated as low (21, 25, 34, 40, 55, 57, 63, 65, 72, 85) and 60 studies (13–20, 22–24, 26–29, 31–33, 35–39, 42, 44–46, 48–54, 56, 58–60, 62, 66, 68–71, 73–84, 86–89) were rated as critically low. A brief overview of the effect sizes of the high quality studies is provided in Table 1.

**Table 1:** Effect sizes of high quality studies.

Type of intervention	Age group	BMIz (MD/SMD, 95% CI)	BMI (MD/SMD, 95%CI)
Treatment			
Diet, physical activity and behavioral (30)	6–11 y	−0.06 (−0.10, −0.02)	−0.53 (−0.82, −0.24)
Prevention			
Diet and physical activity (41)	5-11y	−0.03 (−0.06, 0.00)^ST^ −0.05 (−0.07, −0.02)^MT^ −0.02 (−0.06, 0.01)^LT^	−0.11 (−0.21, −0.01) ^ST^ −0.11 (−0.21, 0.00) ^MT^ 0.03 (−0.11, 0.16)^LT^
Diet and physical activity (43)	2–4 y	−0.03 (−0.07, 0.01)^ST^ −0.07 (−0.11, −0.04)^MT^ −0.07 (−0.13, −0.01)^LT^	−0.08 (−0.20, 0.04) ^ST^ −0.05 (−0.18, −0.08) ^MT^ −0.20 (−0.39, 0.01)^LT^
Diet and physical activity (47)	12-18y	−0.09 (−0.20, 0.02) ^ST^ −0.05 (−0.10, 0.01) ^MT^ −0.02 (−0.05, 0.01) ^LT^	0.03 (−0.07, 0.13) ^ST^ 0.01 (−0.09, 0.11) ^MT^ 0.06 (−0.04, 0.16) ^LT^
Physical activity (61)	6–18 y	−0.06, (−0.09, −0.02)	0.07, (−0.15 to 0.01)
Dietary (64)	3-18y		−0.14 (−0.26, −0.01)
Dietary (67)	6 months to 6 years	−0.03 (−0.09 to 0.03)	−0.08; (−0.23 to 0.07)

ST, Short Term; MT, Medium Term; LT, Long Term.

## Discussion

This review synthesized evidence on the effectiveness of interventions aimed at preventing and managing overweight and obesity among children and adolescents. To better understand the available evidence and existing research, the included reviews were categorized into five broad interventions domains: i. dietary, ii. physical activity, iii behavioral/educational, iv. digital health and v. nutraceutical, with interventions assessed either independently or in combination for prevention, management or both. However, this approach generated a large and heterogeneous body of evidence. Drawing definitive conclusions was challenging owing to the diversity of outcomes assessed and the inconsistent findings reported across individual reviews (90). Nevertheless, interventions combining dietary, physical activity, and behavioral components consistently demonstrated the most promising results. The findings further suggested that interventions delivered during early childhood may be more effective in preventing and managing overweight and obesity than those implemented during late childhood or adolescence. This could be probably owing to the inculcation of healthy dietary and physical activity patterns due to greater parental influence over children’s health behaviors, with increasing difficulty in modifying the established behaviors during adolescence. These observations underscore the need for prioritizing early-life interventions for prevention and management of obesity. Further, the evidence from systematic reviews with high methodological quality further reinforced the effectiveness of combined dietary, physical activity, and behavioral interventions as strategies for preventing and managing overweight and obesity. Collectively, the evidence indicates that multicomponent interventions integrating dietary modification, physical activity, and behavioral change strategies are likely to be effective. These findings are consistent with previous evidence reported elsewhere (91, 92), although the substantial heterogeneity in intervention strategies should be acknowledged. Reviews evaluating dietary, physical activity, and behavioral interventions, whether delivered independently, in combination, or as components of multicomponent programs, incorporated considerable variation in implementation strategies with varyin intervention intensity, duration, setting and follow-up periods.

The evidence base further reveals substantial heterogeneity in the implementation strategies across the variety of interventions delivered. Dietary interventions included nutrition education and counseling, modification of dietary habits and behavior, reflecting a lack of uniformity in the operationalization of diet-related strategies. Regarding physical activity, evidence from two reviews (55, 56) noted the importance of combining aerobic exercise with strength/resistance training for the management of obesity among children. In contrast to this, prevention focused physical activity interventions, were predominantly delivered particularly in school-based settings and emphasized on training, guidance and support for physical activity promotion. These findings suggest that structured and supportive institutional frameworks may be particularly relevant for preventive interventions, whereas targeted high-intensity or combined exercise approaches may be more effective for obesity management.

Considerable variability was also observed for behavioral change intervention strategies which were broadly categorized as social support-based approaches, youth theory-based participatory interventions, motivational interviewing, cognitive behavioral therapy etc. Among these, parental social support emerged as the most commonly implemented strategy, underscoring the central role of family involvement in shaping children’s health behaviors. Only one review highlighted involvement of peers (children and youths) in participatory roles for prevention and management of obesity (54). Collectively, these findings highlight the importance of social support systems as efficacious strategies for prevention and management of children and adolescents. Further, digital health interventions implemented independently or in combination with physical activity demonstrated mixed effects. Interventions involving wearable devices and game- based interventions showed encouraging results, whereas standalone eHealth and mHealth intervention demonstrated limited effectiveness. These observations suggest the potential of digital health approaches.

Environmental determinants, such as school policies, school ambience and the broader built environment with social support systems from family and friends are also likely to play a critical role in shaping behaviors related to overweight and obesity. However, evidence in this domain remained limited, with only a few studies, primarily derived from a sub group analysis. This makes it difficult to assess the effects of these factors on overweight and obesity treatment and prevention in a comprehensive manner. The gap highlights the need for more focused research studies on environmental influences with robust study designs and comparable intervention frameworks. The discussion here underscores the importance of the modifications in environmental conditions and policy-level interventions at the school and the community level as essential to effectively address the problem of overweight and obesity in children and adolescents. Creating supportive school environments, improving access to healthy foods, and promoting opportunities for physical activity, alongside strengthening social support systems, including active involvement of parents and peer groups are likely to complement individual-level interventions and contribute to more sustainable, population-level impact. In the nutshell, the above discussion highlights a significant heterogeneity in the reviews owing to the differences in the selection of the study population as well as the intervention type and intensity, its setting, duration of intervention implementation, follow up period and the outcomes assessed to assess overweight and obesity. This heterogeneity is likely to result in differences in the effect estimates and therefore conflicting conclusions.

Energy homeostasis is regulated by leptin and ghrelin, which are in turn integrated in the hypothalamus to control appetite. Therefore, isolated interventions (e.g., diet alone) often trigger compensatory responses including increased hunger and reduced energy expenditure (93). Physical activity interventions help in preserving lean mass, sustain resting metabolic rate, and improve insulin sensitivity, while also enhancing adipose tissue function and reducing chronic inflammation (94). Further, behavioral strategies like goal setting and self-monitoring delivered within the framework of cognitive behavioral therapy, focus on restructuring thoughts associated with diet and physical activity (95). Digital tools like mobile or e health applications, wearable devices etc. incorporating prompts, social support features, enhance sustained engagement and address barriers associated with low motivation, thereby offering scalable and personalized approaches to behavior change (96). A key strength of this review is the use of the AMSTAR 2 tool for assessing methodological quality of the included systematic reviews. Nevertheless, a substantial proportion of the included reviews were rated as low or critically low quality. Most of the methodological flaws within the reviews could be prevented by pre-registering and adhering to a pre-defined protocol, transparency regarding justifications, implementing a comprehensive and reproducible search strategy, and using standardized tools for risk of bias assessment. These limitations highlight the need for more rigorous and transparent systematic review methodologies. Further, owing to the variations in methodological quality of the reviews assessed using the AMSTAR 2 tool the overall certainty and credibility of the evidence warrants cautious interpretation.

A streamlined search strategy, an accelerated review process along with reliance on a single database (i.e., PubMed) may have resulted in the omission of relevant studies are some of the limitations of this review. Additionally, the possibility of publication bias cannot be ignored. Studies reporting significant or favorable outcomes are more likely to be published and subsequently included in systematic review thereby influencing the overall body of evidence synthesized in the present review. Therefore, the findings of the current review should be interpreted taking into consideration the limitations highlighted and the quality of the studies assessed.

## Conclusion

This rapid review highlights that multicomponent interventions integrating dietary, physical activity, and behavioral strategies are the most consistently effective approaches for the prevention and management of overweight and obesity among children and adolescents. Interventions delivered during early childhood and within supportive family or school environments appear particularly promising. However, the overall evidence base is limited by the low methodological quality and substantial heterogeneity of the included systematic reviews. Future research should prioritize rigorous, high-quality multicomponent studies incorporating environmental and contextual determinants to strengthen the evidence base and support sustainable, scalable public health interventions.

## Footnotes

1
https://www.rayyan.ai/
