# Supplementary material for: Public health interventions for the prevention and management of overweight and obesity in children and adolescents: a rapid review
Source: Front Public Health. 2026 Sep 8;14:1893709. doi: 10.3389/fpubh.2026.1893709 (PMC13595927; doi:10.3389/fpubh.2026.1893709)
Supplement: Supplementary file 1 [file Data_Sheet_1.DOCX]

**Search Strategy for Review of Reviews focusing on prevention and or management of obesity/overweight among children & adolescent (all interventions included)**

("child" OR "children" OR "adolescent" OR "teen" OR "youth" OR "young people" OR "school-aged" OR "school children" OR "school students" OR "minors")

AND

("obesity" OR "overweight" OR "childhood obesity" OR "pediatric obesity" OR "excess weight" OR "body fat" OR "body weight" OR "body mass index" OR "BMI" OR "abdominal obesity" OR "central obesity" OR "adiposity" OR "skinfold thickness" OR “BAZ Score” OR "BMI for age z scores” OR “Body Fat percentage”)

AND

("diet" OR "nutrition" OR "dietary intervention" OR "dietary modification" OR "healthy diet" OR "balanced diet" OR "meal planning" OR "nutrition education" OR "diet counseling" OR "food intake" OR "diet quality" OR "diet supplementation" OR "physical activity" OR "exercise" OR "aerobic exercise" OR "resistance training" OR "strength training" OR" yoga" OR "sports" OR "fitness program" OR "motor activity" OR "movement-based intervention" OR "lifestyle intervention" OR "behavioral intervention" OR "healthy lifestyle" OR "behavior change" OR "screen time reduction" OR "sedentary behavior" OR "sleep" OR "sleep duration" OR "sleep hygiene" OR "stress" OR "stress management" OR "mindfulness" OR "emotional wellbeing" OR "mental wellbeing" OR "motivational interviewing" OR "counseling" OR "self-monitoring" OR "goal setting" OR "digital intervention" OR "mobile app" OR "telehealth" OR "health coaching" OR "environmental modification" OR "community-based intervention" OR "family-based intervention" OR "school-based intervention" OR "public health intervention" OR "health promotion" OR "policy intervention" OR "education intervention" OR "social marketing" OR "health communication" OR “HFSS consumption” )

AND

("prevention" OR "management" OR "treatment" OR "obesity prevention" OR "obesity management" OR "weight management" OR "weight reduction" OR "BMI reduction" OR "body composition" OR "adiposity reduction" OR “Body Fat percentage reduction”)

AND

("systematic review" OR "meta-analysis")
